# Downregulation of microRNA-409-3p promotes aggressiveness and metastasis in colorectal cancer: an indication for personalized medicine

**DOI:** 10.1186/s12967-015-0533-x

**Published:** 2015-06-18

**Authors:** Mulin Liu, Aman Xu, Xiao Yuan, Qiao Zhang, Taotao Fang, Wenbin Wang, Chenglong Li

**Affiliations:** Department of Gastrointestinal Surgery, The First Affiliated Hospital of Bengbu Medical College, Bengbu, 233030 China; Department of General Surgery, The Fourth Affiliated Hospital of Anhui Medical University, Hefei, 230000 China; Department of Vascular Surgery, The Second Affiliated Hospital of Soochow University, Suzhou, 215000 China

**Keywords:** miR-409-3p, CRC, NLK, Tumorigenesis

## Abstract

**Background:**

MicroRNAs play an ess ential role in colorectal cancer development and progression. Aberrant miR-409-3p expression has been reported in several cancers. However, the clinical significance and functions of miR-409-3p in human CRC were not entirely clear.

**Methods:**

miR-409-3p expression levels were determined in 45 pairs of primary CRC and their corresponding adjacent non-tumor tissues by qPCR. The effects of ectopic expression of miR-409-3p on CRC cells proliferation, wound healing, metastasis were investigated by CCK-8, transwell assay and peritoneal spreading nude mice model.

**Results:**

Statistical analysis of clinical cases revealed that low miR-409-3p expression had inclinations towards lager tumor size and local invasion. Ectopic expression of miRNA mimics suggested that miR-409-3p could inhibits the abilities of proliferation, wound healing, metastasis and invasion in CRC cells. Notably, we found the NLK could be a potential target of miR-409-3p.

**Conclusion:**

Our results suggest that miR-409-3p functions as a tumor suppressor by inhibiting the development and metastasis of CRC, suggesting that miR-409-3p is expected to become a new diagnostic marker and a new target of the treatment of CRC.

## Background

Colorectal carcinoma (CRC) is one of the prevalent cancer types, ranking the third of all cancer-related deaths worldwide. Its incidence increases with the change of life style and dietary structure [1], and the metastasis is the major cause of deaths for CRC patients [2]. Although many related genes have been identified in CRC [3], the complicated molecular mechanism of CRC development and progression has not been well understood.

MicroRNAs are non-coding small RNAs that regulate gene expression at the post-transcriptional level, and they are involved in many biological signaling pathways [4]. miRNAs can bind to sequences in 3′-UTR of their targets and induce mRNA degradation or translational repression [5]. Many studies have reported significant difference of microRNAs expression profiling between tumor cells and cells derived from normal tissues, indicating that microRNAs have played important roles in tumorigenesis [6, 7]. MicroRNA-409 dysregulation has been detected in many cancers, including gastric cancer [8, 9], prostate cancer [10] and bladder cancer [11]. However, the function of the miRNA in colon cancer is largely unknown. A recent study demonstrated that miR-409-3p could be used for early detection of CRC [12]. Therefore, we focused on miR-409-3p to further investigate its association with CRC.

In this study, we analyzed the down-regulation expression of miR-409-3p in CRC tissues compared with patient-matched normal tissues, and determined that the expression level of miR-409-3p was closely correlated with the clinicopathologic variables of CRC. Ectopic expression of miR-409-3p inhibits proliferation, invasion and metastasis of CRC cells, reduces the tumorigenic ability of CRC cells in nude mice. We also show that miR-409-3p may function as an tumor suppressor by directly targeting NLK. Thus, our results suggest that miR-409-3p is a potential diagnostic marker and therapeutic target of CRC.

## Methods

### Cell culture

Human colorectal cancer cell lines, SW480 and SW1116, were purchased from American Type Culture Collection (ATCC). SW480 and SW1116 were cultured in Leibovitz’ L-15 Medium (Corning Cellgro^®^, Manassas, VA, USA) with 10% fetal bovine serum (FBS) (Invitrogen, Carlsbad, CA, USA) and 2 mM l-glutamine. Cells were maintained at 37°C/5% CO_2_ in a humidified incubator.

### RNA isolation and qRT-PCR

Total RNA was extracted from cell lines and tissue samples using a mirVana™ miRNA Isolation Kit (Applied Biosystems, Foster City, CA, USA) according to the manufacturer’s instructions. Concentrations and purity of the RNA samples were measured by electrophoresis and spectrophotometric methods. The expression level of miR-409-3p in cell lines and tissue samples was assayed by qRT-PCR and calculated.

### Transient transfection of miRNA mimics

miRNA-409-3p mimics and negative control mimics were purchased from GenePharma (Shanghai, China). Cells were seeded into cell culture plates 20 h before transfection to ensure 70% cell confluence at the moment of transfection. Transfection of miRNA mimics into CRC cells was carried out using Lipofectamine2000 (Invitrogen, Carlabad, CA, USA) according to the manufacturer’s procedure. The miRNA mimics worked at the final concentration of 100 nM. At 48 h post-transfection, qPCR and Western blot were performed.

### Cell proliferation assay

Cell proliferation was assessed by WST (water-soluble tetrazolium salt) assay using the Cell Counting Kit-8 (Dojindo, Kumamoto, Japan) and measured as described in the manufacturer’s instruction. At 24 h post-transfection with mimics or control mimics, CRC cells were seeded onto 96-well plates (2 × 10^3^ cells/well), and cell proliferation was documented every 24 h for 4 days. The number of viable cells was assessed by measurement of the absorbance at 450 nm.

#### Wound healing assay

For scratch wound healing assay, cells were cultured in serum-free medium for 24 h and wounded with pipette tips. Fresh medium was replaced. The wound closing procedure was observed for 48 h, and photographs were taken every 24 h.

### Colony formation assay

In plate colony formation assay, cells were resuspended in RPMI 1640 containing 10% FBS and layered onto 6-well plates (5 × 10^2^ cells/well). The cells were incubated for 2 weeks and stained with crystal violet. Colonies containing 50 cells or more were counted.

### Cell migration and invasion assay

Cell migration and invasion assay was performed using transwell chamber (8 μm, 24-well format; Corning, Lowell, MA, USA). In migration assay, Cells (1 × 10^5^) in 0.2 ml of serum-free medium were added to the upper chamber, and 0.6 ml of medium containing 10% FBS were added to the lower. Cells were then incubated for 24 h. For invasion assay, diluted Matrigel (BD Biosciences) was used to coat the insert chambers’ membrane. Cells were cultured for 48 h under the same conditions. Finally, cells that migrated or invaded into the lower chambers were fixed with methanol, stained with crystal violet and counted in six random fields.

### In vivo metastasis peritoneal spreading assay

CRC cells were resuspended and injected intraperitoneally (2 × 10^6^ cells/mouse) into 4-week-old male BALB/C nude mice (Institute of Zoology, Chinese Academy of Sciences of Shanghai). Five mice were enrolled in each group. 8 weeks after intraperitoneal injection, mice were euthanized by cervical dislocation, and peritoneal spreading of tumor lesions was assessed by necropsy. All experiments were performed in accordance with the official recommendations of the Chinese Animal Committee.

### Statistical analysis

The relationship between the miR-409-3p expression level and clinicopathologic parameters was analyzed by the Person ${\chi^ 2}$ test. The differences between groups were analyzed using Student *t* test. All statistical analyses were performed using the SPSS 13.0 software (SPSS Inc, Chicago, IL, USA), and *P* < 0.05 was considered significant.

## Results

### The expression of miR-409-3p is down-regulated in CRC and correlated with tumor size, and local invasion

**Figure 1:**
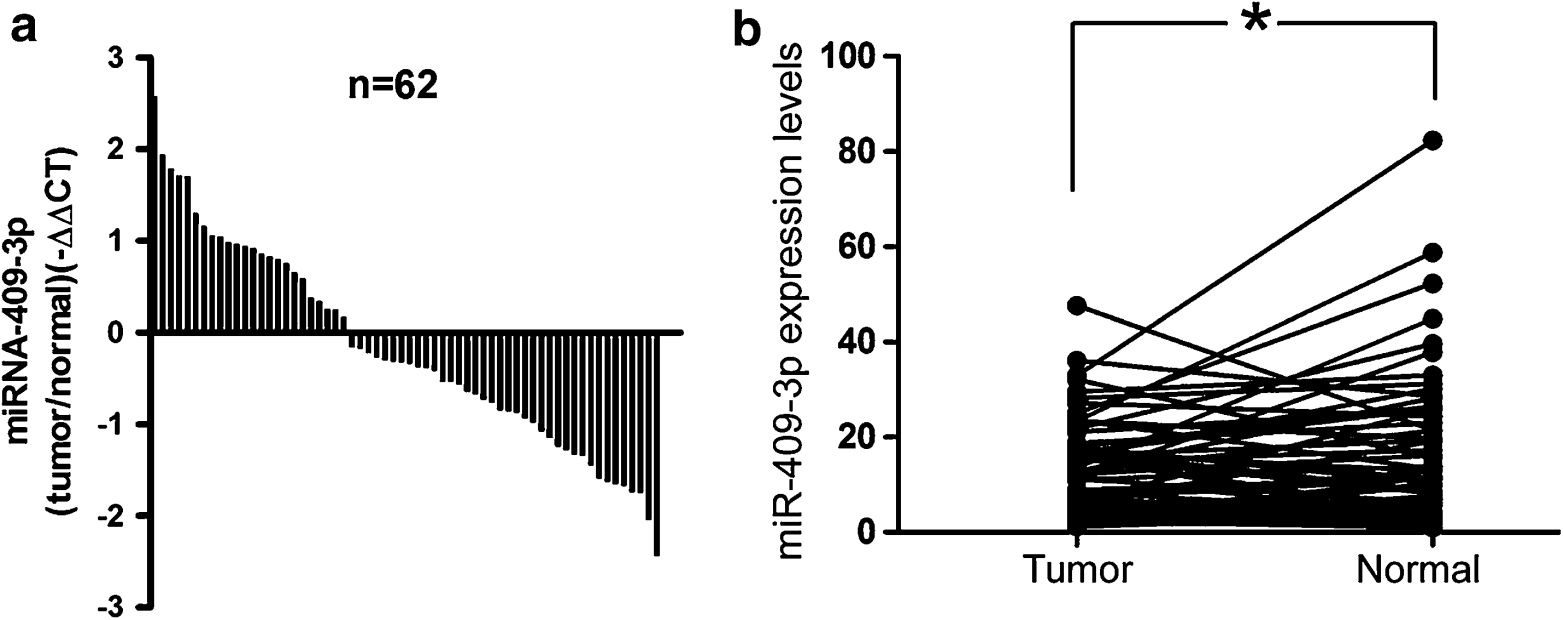
miR-409-3p is downregulated in CRC tissues. **a** Relative expression of miR-409-3p in 45 CRC patients tumor tissues compared with adjacent non-tumor tissues. qRT-PCR results are shown as $^{ - \Delta \Delta} {{\text{CT}}}$ values. **b** Expression levels of miR-409-3p in 45 CRC patients were analyzed by qRT-PCR. Data is shown as ${2^{ - \Delta {\text{Ct}}}}$ (*P < 0.05).

To explore the expression levels of miR-409-3p in CRC tissues and adjacent non-tumor tissues, qRT-PCR analysis was performed. 45 CRC paired samples were included in this study. The results showed that the average expression level of miR-409-3p was significantly down-regulated in tumor tissues compared to the adjacent non-tumor tissues (Figure 1a, b). Furthermore, we elucidated the correlation between miR-409-3p expression and clinicopathologic factors. As shown in Table 1, we also evaluated the relationship between miR-409-3p expression levels and its clinicopathologic characteristics in 45 CRC patients. Results showed that the low expression of miR-409-3p was significantly correlated with tumor size and local invasion (*P < 0.05). Whereas, miR-409-3p level was not associated with other clinicopathologic features, including age, sex (*P* > 0.05).

**Table 1:** Relationship between miR-409-3p expression levels and clinicopathological variables in 62 CRC patients

Clinicopathologic parameters	No. of patients	miR-409 expression		*P* value
		Low (n = 38)	High (n = 24)	
Age (years)				0.180
<60	27	14	13	
≥60	35	24	11	
Gender				0.173
Male	32	17	15	
Female	30	21	9	
Tumor size (cm)				0.027*
<5	33	16	17	
≥5	29	22	7	
Local invasion				0.011*
T1, T2	13	4	9	
T3, T4	49	34	15	
Lymph node				0.118
N0	31	16	15	
N1–N3	31	22	9	
Metastasis				0.702
M0	53	33	20	
M1	9	5	4	

*Statistically significant *P* values (*P* < 0.05)

### miR-409-3p inhibits CRC cells proliferation and clonogenicity of CRC cells in vitro

**Figure 2:**
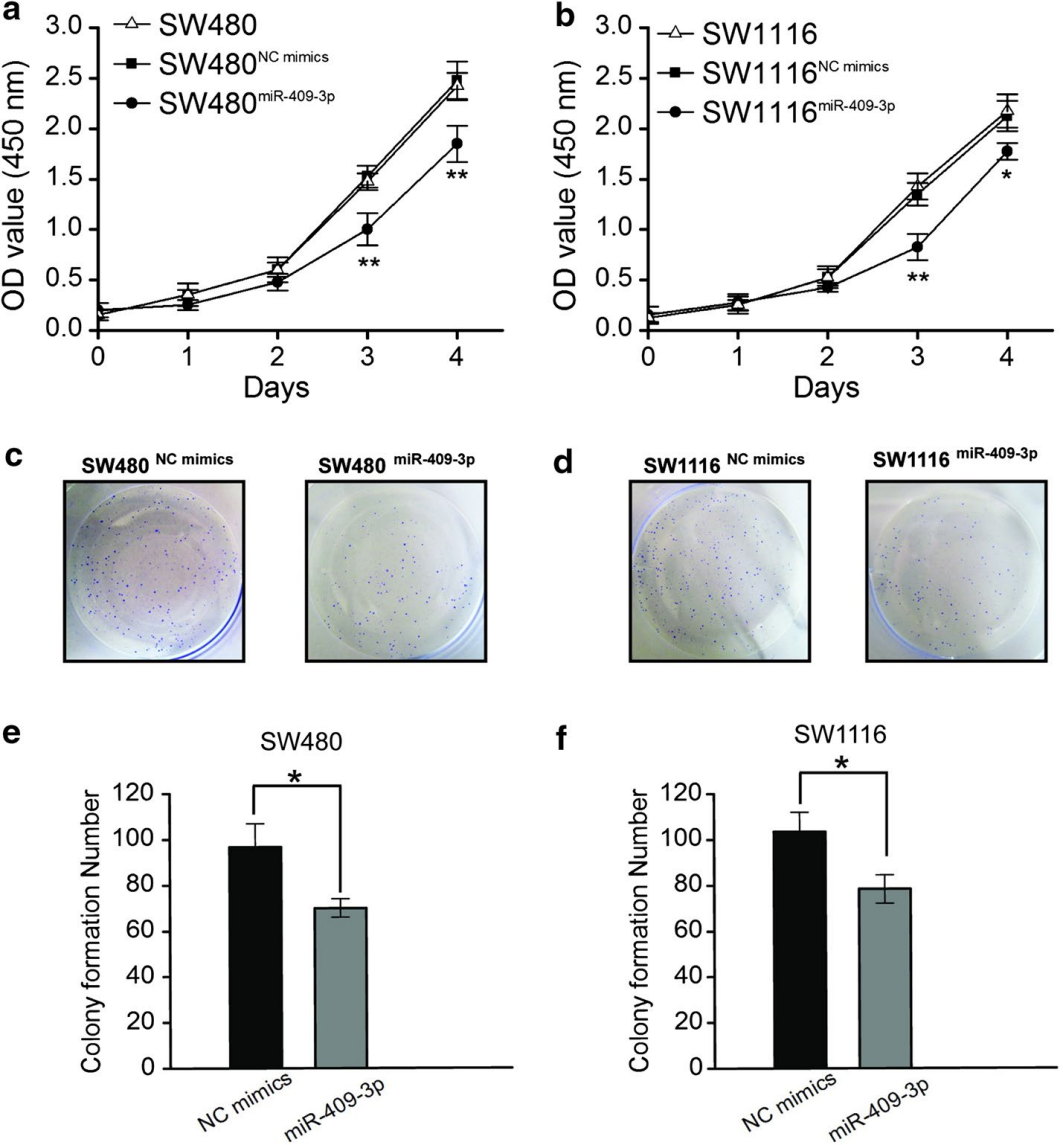
The effect of miR-409-3p on the proliferation of CRC cells. **a** Cell proliferation was measured by the WST assay. SW480 cells were transfected with miR-409-3p mimics or NC mimics at a final concentration of 100 nM and, at 24 h post-transfection, the WST assay was performed every 24 h for 4 days. Results aremeans of three independent experiments ± SD (**P < 0.01). **b** SW1116 cells were transfected with miR-409-3p mimics or NC mimics at a final concentration of 100 nM and, at 24 h post-transfection, the WST assay was performed every 24 h for 4 days. Results are means of three independent experiments ± SD. **c** The effect of miR-409-3p on cell proliferation was evaluated by the plate colony formation assay. SW480 cells were transfected with miR-409-3p mimics or NC mimics then seeded onto 6-well plates. The number of colonies was counted on the 14th day after seeding. **d** SW1116 cells were transfected with miR-409-3p mimics or NC mimics then seeded onto 6-well plates. The number of colonies was counted on the 14th day after seeding. **e**, **f** Colonies containing 50 or more cells were counted. Results are means of three independent experiments ± SD (*P < 0.05).

Considering that miR-409-3p is significantly down-regulated in CRC, it may function as a tumor suppressor. Therefore, we next determined whether overexpression of miR-409-3p in CRC cells could affect cell proliferation. miR-409-3p mimics or control mimics oligos were transfected into CRC cells respectively. We examined the effects of miR-409-3p on cell growth by WST cell-growth assay. The results showed that cell growth was inhibited in cells transfected with miR-409-3p mimics compared with the cells transfected with control mimics (Figure 2a, b). To further characterize the effect of miR-409-3p on cell proliferation, the colony formation assay was performed. The results showed that the number of colonies from CRC cells transfected with miR-409-3p mimics was significantly fewer than that from the control group (Figure 2c, d). The number of colonies from SW480^miR-409-3p^ and SW1116^miR-409-3p^ cells was fewer than that from the control groups (SW480^NC mimics^ and SW1116^NC mimics^, *P < 0.05, Figure 2e, f). These findings indicate that miR-409-3p inhibits the proliferation and colony forming ability of CRC cells.

### miR-409-3p inhibits scratch wound healing ability of CRC cells

**Figure 3:**
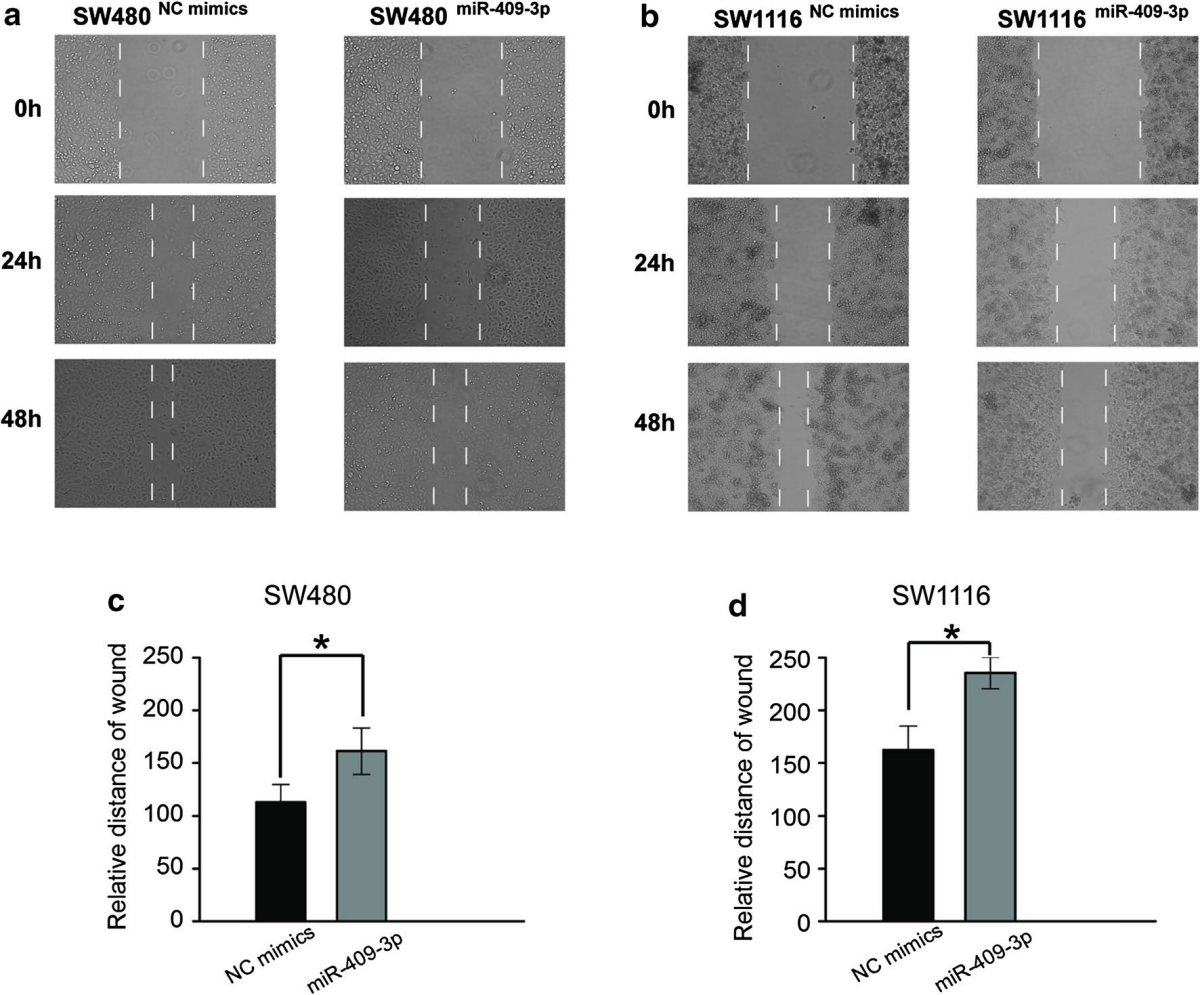
miR-409-3p inhibits scratch wound healing ability of CRC cells. **a** Movement ability of SW480^NC mimics^ and SW480^miR-409-3p^ cell lines was detected by scratch wound healing assays. **b** Movement ability of SW1116^NC mimics^ and SW1116^miR-409-3p^ cell lines was detected by scratch wound healing assays. By wound distance analysis, the movement ability of CRC cells was inhibited by miR-409-3p.

We detected whether miR-409-3p can change the movement ability of CRC cells by scratch healing assay. The results were shown in Figure 3a, miR-409-3p overexpression cell lines SW480^miR-409-3p^ restoration slowly closed the scratch wounds compared with the control 48 h after scratching, in contrast to the control SW480^NC mimics^ cells which were significantly efficient in wound healing. The results in SW1116 cells groups are consistent with above data (Figure 3b).

### miR-409-3p inhibits migration and invasion of CRC cells in vitro

**Figure 4:**
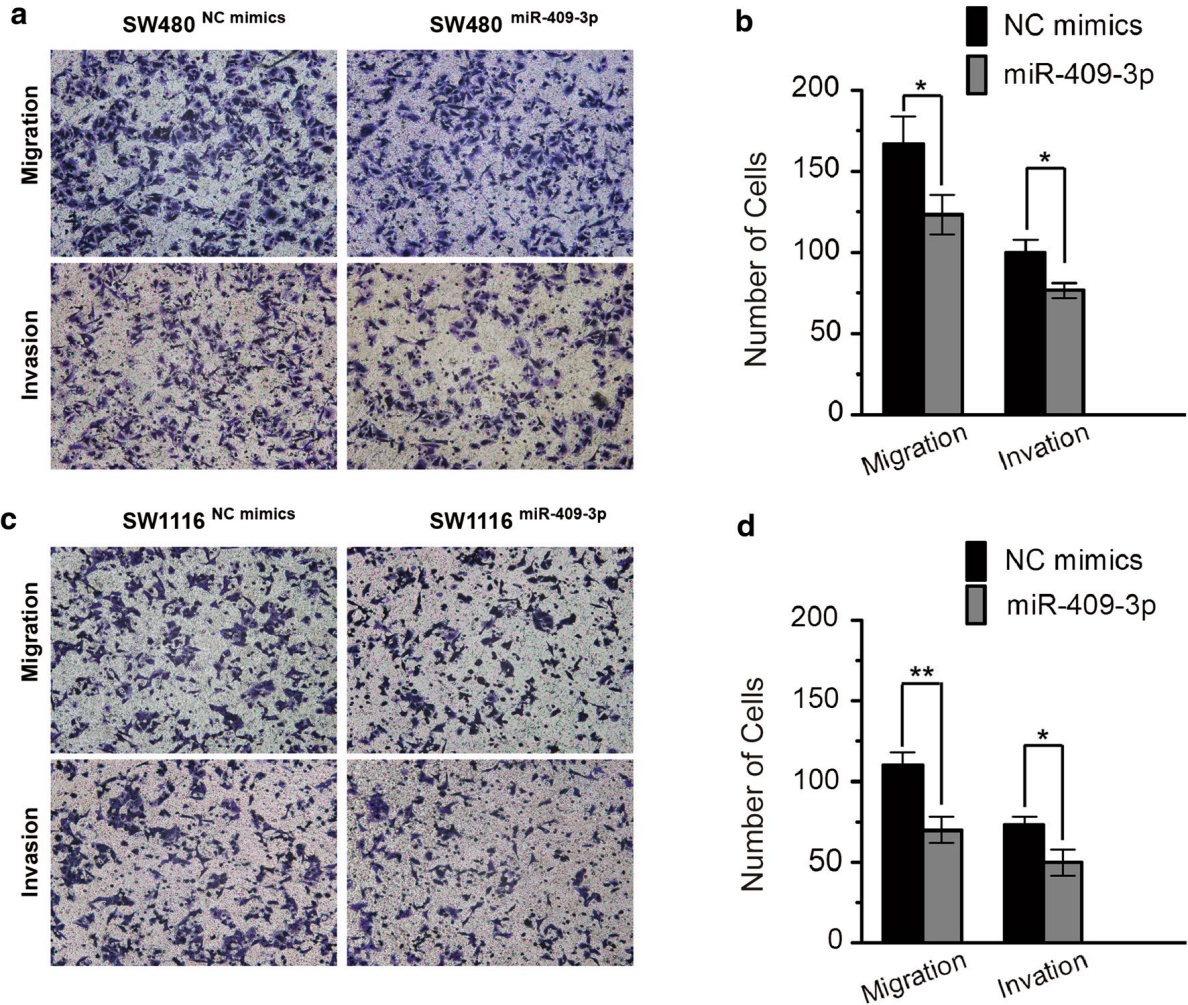
miR-409-3p inhibits migration and invasion of CRC cells. **a** Representative photographs of migratory or invasive SW480^NC mimics^ and SW480^miR-409-3p^ cells on the membrane (magnification, ×100). **b** Average number of migratory or invasive SW480^NC mimics^ and SW480^miR-409-3p^ cells. (*P < 0.05). **c** Representative photographs of migratory or invasive SW1116^NC mimics^ and SW1116^miR-409-3p^ cells on the membrane (magnification, ×100). **d** Average number of migratory or invasive SW1116^NC mimics^ and SW1116^miR-409-3p^ cells (*P < 0.05, **P < 0.01). The data represent the mean ± SD of three independent experiments.

In order to further assess the influence of miR-409-3p on CRC cells, we further explored its effects on cell migration and invasion, a key ability of tumor progression and metastasis. The results showed that overexpression of miR-409-3p led to significantly inhibited migration and invasion of CRC cells. As shown in Figure 3, the number of migratory and invasive SW480 cells transfected with miR-409-3p mimics was significantly less than the control group (*P < 0.05. Figure 4a, b), and number of migratory and invasive SW1116 cells transfected with miR-409-3p was significantly less compared to the control group (*P < 0.05, **P < 0.01. Figure 4c, d).

### miR-409-3p induces CRC cells apoptosis

**Figure 5:**
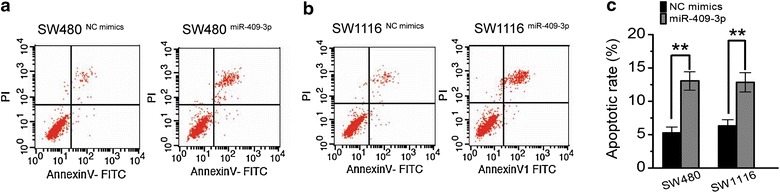
miR-409-3p induces CRC cells apoptosis. **a** Representative histograms depicting apoptosis of SW480 cells transfected with miR-409-3p mimics or control. **b** Representative histograms depicting apoptosis of SW1116 cells transfected with miR-409-3p mimics or control. **c** The percentage apoptotic cells of three independent experiments ± SD (**P < 0.01).

To investigate the mechanism of miR-409-3p on CRC cell proliferation inhibitory effects, we performed apoptosis analysis by flow cytometry. The results indicated that apoptotic rate was significantly increased in miR-409-3p mimics transfected groups compared to the control groups both in SW480 and SW1116 cells (Figure 5). The results supported that miR-409-3p could be a potential therapeutic target.

### miR-409-3p inhibits peritoneal spreading CRC cells in nude mice in vivo

**Figure 6:**
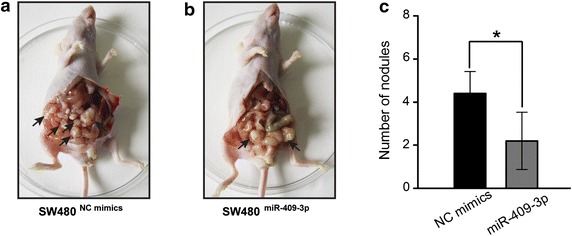
miR-409-3p inhibits peritoneal spreading in nude mice. **a**, **b** Representative images of nude mice injected with indicated cells. **c** Quantification of the peritoneal nodules is shown in the *bar graph*. The results are mean ± SD, *P < 0.05.

Given that miR-409-3p inhibits metastasis of CRC cells in vitro, we next tested whether ectopic expression of miR-409-3p could influence the tumor growth and metastasis of CRC cells in vivo. To confirm the contribution of miR-409-3p in vivo, we carried out peritoneal spreading in mice models. CRC cells transfected with miR-409 mimics or control mimics were selected and intraperitoneally injected into nude mice, These mice were euthanized 8 weeks after the injection, and the tumor lesions in the peritoneal cavity were counted. Strikingly, the peritoneal nodules were significantly inhibited in miR-409-3p mimics transfected group compared with control group (Figure 6). Thus, these results indicate that miR-409-3p could suppress metastasis of CRC cells in vivo.

### miR-409-3p targets the 3′-UTR of *NLK*

**Figure 7:**
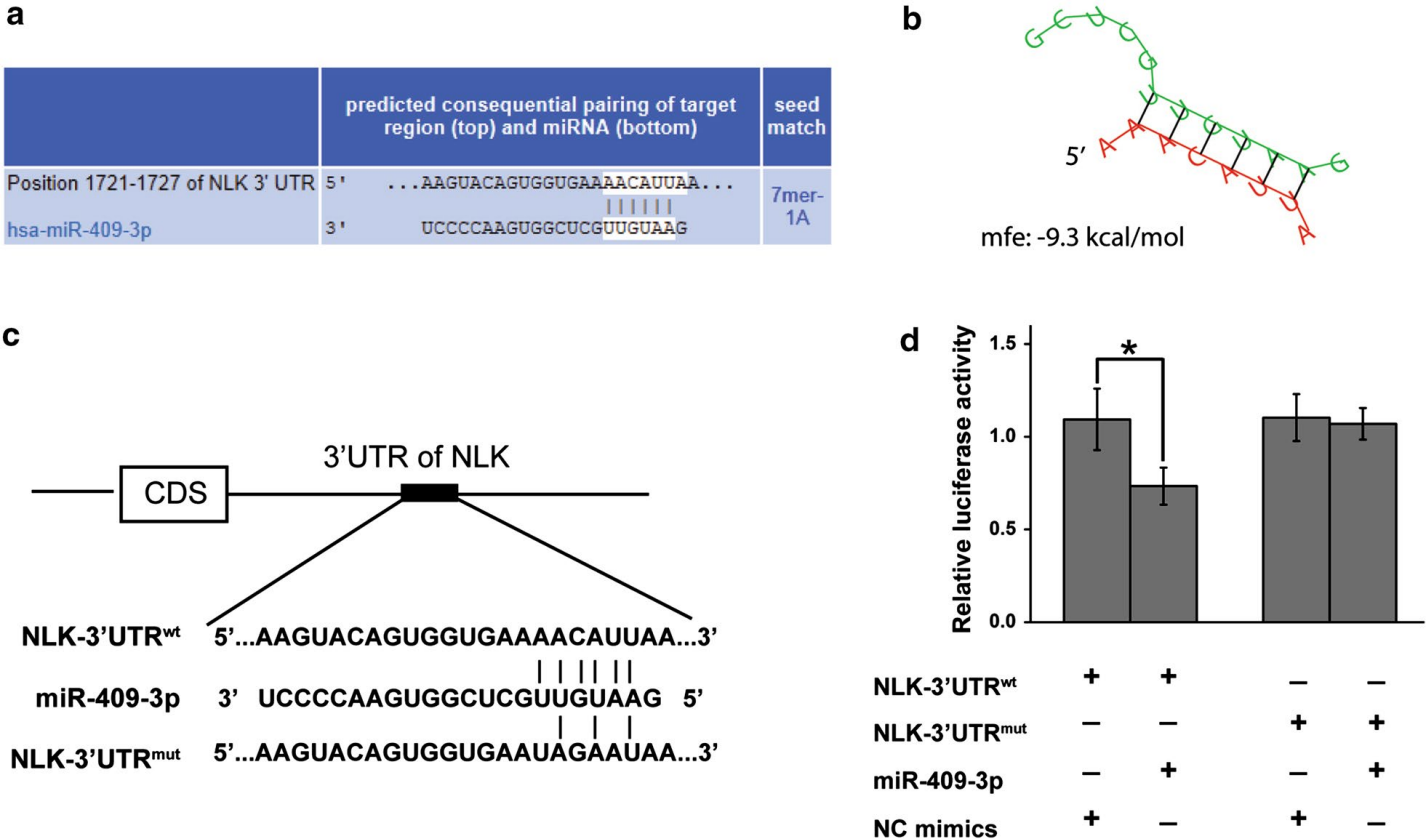
miR-409-3p targets the 3′-UTR of the oncogene *NLK.* **a** The putative binding sites of miR-409-3p in *NLK* 3′-UTR region were predicted. The matched seed sequences were indicated by *vertical lines*. **b** The minimum free energy (mfe) required for RNA hybridization was predicted by RNAhybrid software (mfe: −9.3 kcal/mol). **c** Schematic graph of the putative binding sites of miR-409-3p in the *NLK* 3′UTR and the mutation in miR-409-3p-binding sites. **d** miR-409-3p mimics down-regulated luciferase activities controlled by wild-type *NLK* 3′UTR (*P < 0.01), but did not affect luciferase activity controlled by mutant *NLK* 3′UTR. The results are means of three independent experiments ± SD.

To identify how miR-409-3p functions in CRC cells, computational prediction of miR-409-3p targets was performed. We searched miR-409-3p’s putative targets using online search tools (e.g. TargetScan, Microrna.org and RNAhybrid) [13–17]. The genes predicted by all the used programs were considered candidate targets of miR-409-3p. Among all the hits, NLK caused our attention (Figure 7a, b). We then performed luciferase reporter assays to verify a direct interaction between miR-409-3p and the 3′UTR of *NLK*. Luciferase reporters were constructed containing either a wild-type *NLK* 3′UTR sequence containing the miR-409-3p binding site (NLK-3′UTR^wt^), or a mutated 3′UTR (NLK-3′UTR^mut^) (Figure 7c). The relative luciferase activity of the NLK-3′UTR^wt^ reporter was markedly suppressed by miR-409-3p mimics compared with that of NLK-3′UTR^mut^ in a miR-409-3p dependent manner (Figure 7d). This result strongly indicates that 3′UTR of *NLK* carries the direct binding seed of miR-409-3p.

## Discussion

In recent years, many studies have confirmed the key role of miRNAs in CRC. Although the signatures of miRNAs on CRC have been well characterized, the functions of dysregulated miRNAs in CRC progression and development remains unclear. In pervious studies, dysregulation of miR-409-3p has been reported in many cancers, including gastric cancer [8, 9], prostate cancer [10] and bladder cancer [11]. In these studies, the expression levels of miR-409-3p was significantly lower in cancer tissues compared with corresponding non-tumor tissues, and it inhibits proliferation, invasion and tumorigenesis in vitro or in vivo, suggesting that it may serve as a tumor suppressor. miR-409-3p has not yet been well explained in CRC.

In the present study, statistical analysis of clinical cases revealed that low miR-409-3p expression had inclinations towards lager tumor size and local invasion. The results suggested that the functions of miR-409-3p in CRC were worthy to study further. Ectopic expression of miR-409-3p mimics suggested that miR-409-3p could inhibits the abilities of proliferation, wound healing, metastasis and invasion in CRC cells. The peritoneal spreading nude mice assay confirmed that miR-409-3p could inhibits the ability of metastasis in CRC cells in vivo, which is an important aspect of tumor development.

MiRNAs have been usually recognized as regulatory factors through decreasing translation of target mRNA or increasing degradation of target mRNA [18]. It has been reported that miR-409-3p targets PHF10, radixin and c-Met [8, 9, 11, 19] Here, we found that NLK could be a potential target of miR-409-3p since NLK would be the potential target in the CRC scenario.

NLK, an evolutionarily conserved serine/threonine protein kinase [20], is involved in many important biological processes and signaling pathways, such as Wnt/β-catenin pathway. This pathway and its downstream components have a key role in cancer progression and development through many processes including tumor initiation, proliferation and metastasis [21]. The role of NLK in different tumors was incoherent. NLK was presumed to be a tumor suppressor gene in ovarian cancer, glioma and prostate cancer [22–24]. While in gallbladder cancer, hepatocellular carcinoma and laryngeal cancer, NLK functioned as oncogene [25–27]. In a recent study, the expression levels of NLK were significantly upregulated in CRC tissues compared to which in matched non-tumor tissues, and the NLK expression was significantly correlated with the clinicopathological features including the depth of tumor invasion [28]. This result is consistent with the expression profile of miR-409-3p in our study. A recent investigation on a large cohort of over 2,000 CRC patients indicated that NLK might promote the aggressiveness of CRC, which is consistent with the expression profile of miR-409-3p in the present study. NLK was essential for CRC tumor initiation but not required for tumor progression in murine CRC model [29]. All these findings suggest that NLK plays an important role in CRC tumorigenesis, but the role in different CRC tumors might be somewhat different. It will be of interest to elaborate the exact role of NLK in human CRC progression under different conditions. As a negative regulator of NLK, miR-409-3p has a naturally potential value in personalized medicine. The combination of protein gene and miRNA has been emerged as a tool to predict the efficacy of treatment [30, 31].

The transcriptional regulation of miR-409-3p has been implicated in some diseases [32, 33]. The transportation of miR-409-3p was implicated in early CRC detection [12]. Therefore, the mechanism underlying the control of miR-409-3p level in colorectal cells is expected to be addressed in the future study.

## Conclusion

In conclusion, our data showed that miR-409-3p was frequently down-regulated in CRC. miR-409-3p functions as a tumor suppressor by inhibiting the development and metastasis of CRC, suggesting that miR-409-3p is expected to become a novel diagnostic marker and a new target of the treatment of CRC.

## Abbreviations

CRC: colorectal carcinoma
NLK: nemo-like kinase
UTR: untranslated region
